# Computer-Vision-Oriented Adaptive Sampling in Compressive Sensing †

**DOI:** 10.3390/s24134348

**Published:** 2024-07-04

**Authors:** Luyang Liu, Hiroki Nishikawa, Jinjia Zhou, Ittetsu Taniguchi, Takao Onoye

**Affiliations:** 1 Graduate School of Information Science and Technology, Osaka University, Osaka 5650871, Japan; liu.luyang@ist.osaka-u.ac.jp (L.L.); zhou@hosei.ac.jp (J.Z.); onoye@ist.osaka-u.ac.jp (T.O.); 2 Graduate School of Science and Engineering, Hosei University, Tokyo 1848584, Japan; nishikawa.hiroki@ist.osaka-u.ac.jp

**Keywords:** compressive sensing, adaptive sampling, data acquisition, computer vision

## Abstract

Compressive sensing (CS) is recognized for its adeptness at compressing signals, making it a pivotal technology in the context of sensor data acquisition. With the proliferation of image data in Internet of Things (IoT) systems, CS is expected to reduce the transmission cost of signals captured by various sensor devices. However, the quality of CS-reconstructed signals inevitably degrades as the sampling rate decreases, which poses a challenge in terms of the inference accuracy in downstream computer vision (CV) tasks. This limitation imposes an obstacle to the real-world application of existing CS techniques, especially for reducing transmission costs in sensor-rich environments. In response to this challenge, this paper contributes a CV-oriented adaptive CS framework based on saliency detection to the field of sensing technology that enables sensor systems to intelligently prioritize and transmit the most relevant data. Unlike existing CS techniques, the proposal prioritizes the accuracy of reconstructed images for CV purposes, not only for visual quality. The primary objective of this proposal is to enhance the preservation of information critical for CV tasks while optimizing the utilization of sensor data. This work conducts experiments on various realistic scenario datasets collected by real sensor devices. Experimental results demonstrate superior performance compared to existing CS sampling techniques across the STL10, Intel, and Imagenette datasets for classification and KITTI for object detection. Compared with the baseline uniform sampling technique, the average classification accuracy shows a maximum improvement of 26.23%, 11.69%, and 18.25%, respectively, at specific sampling rates. In addition, even at very low sampling rates, the proposal is demonstrated to be robust in terms of classification and detection as compared to state-of-the-art CS techniques. This ensures essential information for CV tasks is retained, improving the efficacy of sensor-based data acquisition systems.

## 1. Introduction

With the rise of the Internet of Things (IoT), there has been a trend to acquire and process image data on edge devices for computer vision (CV) tasks. IoT systems often employ a variety of sensors, including cameras, which generate large volumes of image data that require efficient processing and transmission. For example, drones equipped with cameras can be used to explore hazardous areas and outsource their images to other computers [1]. However, the large amounts of raw data generated by these sensor-equipped IoT devices significantly increase the transmission requirements. There is a significant need for efficient techniques that can reduce the transmission cost, enabling effective processing of sensor data such as images in low-bandwidth scenarios [2].

**Figure 1:**
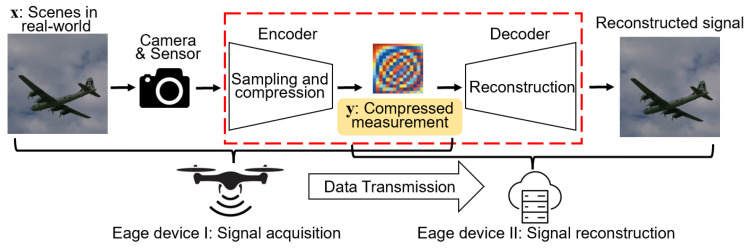
A fundamental concept of CS.

One potential solution is to use compressive sensing (CS) techniques [3], which can efficiently compress the data by requiring much fewer sampling measurements than the traditional Nyquist theory [4], which reduces the transmission cost. The fundamental concept of CS is illustrated in Figure 1. A sensor captures a signal $x\in R^{N}$ of real-world scenes; then, the encoding process results in a compressed measurement $y\in R^{M}$ by signal sampling and compression. Here, *M* and *N* represent the dimensions. Since the signal is sampled and compressed, M<N, and the sampling rate is defined as r=M/N. The sampling rate determines the size of the compressed measurement and, thus, the transmission cost. These compressed measurements are used at the data-receiving end to reconstruct the image, and the obtained reconstructed image can be used for downstream CV tasks. Relevant research has demonstrated that CS-based coding and decoding has faster speed, lower complexity, and better reconstruction quality compared to traditional methods using JPEG, H.264/AVC, or H.265/HEVC standards [5], making it highly beneficial for sensor data processing in IoT systems. By combining CS with CV, it is possible to reduce the amount of data that needs to be transmitted from the sensor data acquisition end to the data processing end.

**Figure 2:**
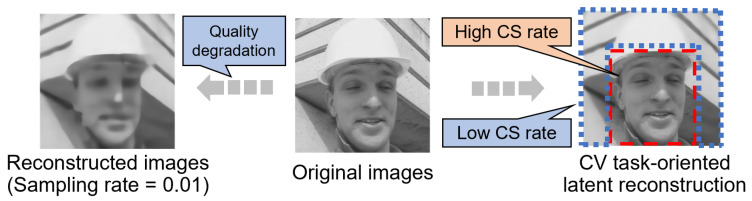
A potential motivation of CV-oriented latent CS [12]. Reproduced with permission from Luyang Liu, *Proceedings of the 5th ACM International Conference on Multimedia in Asia*; published by ACM, 2023.

In recent years, there have been precedents for applying CS technology in the CV field, such as separating sensitive regions of the face from compressed measurements for privacy preservation [6] and proposing backpropagation rules to efficiently localize cells in compressed measurements [7]. However, such existing CS literature has claimed that these methods still suffer from limited applicability in CV tasks since image quality always degrades with a decreasing sampling rate. In the work [8], a block-based CS technique divides the original large-size image into several equal-sized blocks and samples each block at the same sampling rate. This approach greatly reduces the complexity of sampling and became a baseline technique that inspired later research. However, since the whole image is sampled at a uniform sampling rate, the overall quality of the reconstructed image will also inevitably degrade when the sampling rate is reduced, leading to the degradation of the CV inference. Although there are some adaptive sampling CS methods that have been proposed recently, their allocatable sampling rate is still limited to the overall base sampling rate, with the maximum and minimum sampling rates fixed at certain ranges [9,10,11]. Figure 2 depicts an example with different sampling rates and image qualities. If the image is reconstructed at a uniformly low sampling rate, its image is degraded in terms of visual quality, as shown in Figure 2 (left). In addition, the accuracy of inference, such as for classification, should also be degraded when using uniformly low-quality reconstructed images. Here, we come up with the idea that CV tasks may require less information than that necessary to reconstruct an image. By adaptively allocating the sampling rates for blocks, we can achieve high accuracy even at a partially low sampling rate, such as the example in Figure 2 (right), wherein only the region of interest (e.g., the human face) is sampled, while the other regions are ignored.

In this context, the proposed technique contributes to the field of sensing technology and CS by introducing a highly adaptable CV-oriented CS framework that empowers sensor systems to selectively capture and transmit the most relevant data. This paper aims to achieve high inference accuracy in CV tasks such as image classification and object detection. While the existing CS methodologies excel at advancing the quality of image reconstruction, their primary focus is on the visual effect of the final reconstructed image rather than its impact on downstream CV tasks. The main contribution of this paper is to propose a specialized adaptive sampling rate allocation strategy that focuses on specific areas. Past methods always had difficulty with the visual incoherence of an image due to the differences in the sampling rates of each part when using an adaptive strategy. However, this research goes beyond this limited thinking and proposes a CS method that serves only the computer and not the human eye. The proposed method compresses just the information needed for the CV task at a higher sampling rate while discarding non-essential information at a lower sampling rate. This strategy may lead to degradation of the visual effect of the whole image, but the task of creating a reconstructed image that can be recognized correctly can be achieved with less sampling resources overall. In the proposed technique, by employing saliency detection and adaptive sampling, sensors can dynamically assess which data points are most relevant for the task at hand. This intelligent acquisition reduces data overload and ensures that critical information is always given precedence, thereby optimizing the overall data acquisition process. This dynamic acquisition is particularly crucial for computer vision applications, where the quality and relevance of data significantly impact performance and decision-making accuracy. Specifically, the proposed technique first performs saliency detection, and then, it adaptively samples the salient and non-salient blocks at high and low sampling rates, respectively. This saliency feature information plays an important role in subsequent CV tasks, allowing the target to be recognized and, thus, improving inference accuracy. Our technique guarantees that crucial information for CV tasks is preserved, thereby enhancing the effectiveness and efficiency of data acquisition in sensor-based systems. In addition, our technique enables more sophisticated analytics and machine learning models to be applied. This supports more sophisticated data analytics and facilitates more smart applications that use sensors. The goal of our proposal is to find a good allocation of sampling rates in such a way that the inference accuracy of the CV task is improved during sampling rate allocation.

In addition, traditional sensor systems often struggle with the transmission of large volumes of data, which can lead to bottlenecks and inefficiencies. Our technique mitigates this issue by compressing data in a way that retains essential information while discarding non-critical elements. By ensuring that only the most relevant data are prioritized, our technique maintains high data accuracy and quality even at lower sampling rates. The ability to transmit only the most relevant data minimizes the need for extensive data processing and storage infrastructure. This not only lowers operational costs but also extends the lifespan of sensor systems by reducing the computational load and energy consumption required for data processing and transmission. The reduction in data volume translates to lower costs associated with data storage and management. This is particularly beneficial for large-scale sensor networks that generate massive amounts of data. Our technique enables organizations to minimize infrastructure costs while maximizing the utility and effectiveness of their data. This leads to faster data transmission rates, lower bandwidth consumption, and improved real-time data processing capabilities, making it ideal for applications in remote monitoring and IoT networks. The proposed adaptive sampling technique marks an improvement in sensor technology and introduces a paradigm shift in how sensor systems acquire, process, and transmit data. This technique addresses several challenges faced by contemporary sensor networks and enhances their efficiency, effectiveness, and versatility. It paves the way for smarter, more responsive sensor technologies that are better equipped to meet the growing demands of modern applications.

In our previous work [12], we examined the effectiveness of the proposed CS sampling technique in classification tasks. In this paper, to assess the impact of block size on our proposed block-based sampling technique tailored for CV, we compare three different block sizes across multiple classification datasets to discern the resulting variations in accuracy. Furthermore, in order to investigate the broader implications of the proposed technique on diverse CV tasks, this paper applies our proposal to the detection task, thereby expanding the applicability of the CS technique. In order to verify the impact of the proposal on the effectiveness of the utilization of the data collected by the sensors, this paper conducts experiments on datasets collected by a variety of realistic sensors, such as STL10, Imagenette (personal cameras), and Intel (surveillance cameras) for the classification task and KITTI (vehicle camera) for the detection task. Additionally, we provide a comparative analysis with other state-of-the-art CS techniques that are oriented towards image quality.

The proposed technique leverages sensor data more effectively, making it particularly valuable for IoT applications, where data transmission and processing efficiency are crucial. The contributions of this paper are as follows:

- Adaptive sampling for enhanced sensor data utilization: By implementing an adaptive sampling strategy based on saliency detection, our proposal improves the quality and relevance of data collected by sensors. Our proposal effectively preserves essential information that is crucial for CV tasks, leading to more accurate and efficient processing in downstream CV tasks.
- Wide versatility for different CV tasks: To comprehensively evaluate the effectiveness of our proposal, we extend the application from image classification to more intricate objection detection. The experimental results substantiate the superiority of our proposal over existing adaptive sampling techniques. This shows the versatility and broad applicability of our proposal.
- Improvement of CV task accuracy at low sampling rates: Unlike traditional CS techniques that focus on visual quality, our technique enhances the accuracy of CV tasks even at low sampling rates, making it a robust solution for sensor data analysis in real-world scenarios. This highlights a promising solution for maintaining accuracy at a reduced cost of sampled data.

The rest of the paper is organized as follows. Section 2 summarizes recent CS techniques. Section 3 addresses adaptive sampling with saliency detection. Section 4 mentions experiments. Section 5 concludes this paper with future remarks.

## 2. Related Work

The main tasks of CS are to efficiently sample data from the original signal and to accurately reconstruct the signal from the sampled data. The research on CS is divided into two aspects—sampling and reconstruction—and most of the recent work focuses on the sampling part. For example, in [5], the author introduced a low-cost, accurate rate control algorithm based on packet dropping that achieves faster coding and more accurate compression, especially for signal sequences with low and medium motion levels. When the sampling rate is reduced, the amount of data that can be captured and retained from the original signal is also reduced, which inevitably brings about the loss of information, thus causing blurring of the reconstructed image. To correct image degradation due to low sampling rates, the mainstream method is to develop dynamic sampling matrices to enhance the sampling capability. With the development of deep learning, neural-network-based CS techniques have been proposed in order to learn representations of features; these methods have proven to be effective [13]. Zhang [14] proposed a deep learning system for attention-guided dual-layer image compression to form a compact sampling matrix. Fan [15] proposed a global sensing module to collect all level features of an original image in order to reuse measurements multiple times at a multi-scale. Among many types of research, the block-based method, which splits the original image into multiple blocks and samples them simultaneously, improves the efficiency and proves to be effective at processing high-dimensional images [8]. The block-based method has also become a mainstream idea in CS and has enlightened future research.

In the work [16], the authors have noticed that in block-based-sampling CS, different blocks contain different amounts of information: some blocks are richer in texture and detail than others. Therefore, it is significant to adaptively allocate sampling rates to blocks based on information richness. Yu [9] normalized the image to obtain the distribution probabilities of saliency features and then allocated different sampling rates adaptively. Zhou [10] divided the image into asymmetric blocks for fine-grained allocation of sampling rates based on the similarity of the feature values of each part of the image. Converting the original image into a feature distribution map and then allocating different sampling rates according to the feature differences between each block has become the basic process of adaptive CS, which is the idea adopted in this paper. You [17] proposed a framework that solved the problem of the non-uniform size of compressed measurements produced by each block sampled at different sampling rates. This work provides the idea for this paper to realize the simultaneous processing of multiple-sampling-rate sampling by a single model. Chen [18] and Yang [11] applied a content-aware and moving-area-aware scalable network, respectively, to achieve high-quality reconstruction of detailed textures compared to uniform-sampling CS.

However, sampling various blocks of an image with different sampling rates results in the blocking artifact [19]. The blocking artifact means the phenomenon of significant differences between neighboring blocks. To mitigate the deterioration of image quality caused by the blocking artifact, the aforementioned adaptive CS techniques adopt a conservative sampling rate allocation strategy. The works [9,10] limited the allocated maximum and minimum sampling rates to a certain ratio. The authors of [18] fused the reconstructed images using double-sampling to minimize the dissimilarity between blocks, but this resulted in an increase in the cost of sampling and transmission. Since the existing CS techniques take the improvement of image visual quality as the only goal, this leads to the allocation of the sampling rate always needing to consider the visual effect of the whole image as the target. In contrast, our proposed technique takes improving the classification accuracy of the reconstructed images as a goal. The allocation of the sample rate focuses only on the saliency target: that is, the region that may be of interest to the CV task. By separately allocating high and low sampling rates, the overall average sampling rate is reduced while preserving the information needed for classification.

## 3. Proposed CV-Oriented Adaptive Sampling

In this section, we propose a saliency-based block sampling technique in which the inference accuracy of the reconstructed image in CV tasks is improved. Block-based CS has been proven to be effective at handling high-dimensional images by decomposing the original image into a number of equal-sized blocks for simultaneous sampling of each part [20,21,22]. We note that the information in an image is not always uniformly distributed. Therefore, it is necessary to allocate different sampling rates to different blocks depending on the richness of the information. This strategy is called adaptive sampling.

Our proposed adaptive sampling technique seeks to optimize image sampling to specifically enhance the accuracy of downstream CV tasks. Specifically, we determine the distribution of the information by obtaining a feature map of the image. In recent years, neural-network-based saliency detection techniques have been demonstrated to extract global features better than traditional filter transformation methods [23]. According to the definition of saliency detection, in general, locations with low spatial correlation with their surroundings are salient [24]. Based on that, we can localize the salient and non-salient blocks in the image and allocate different sampling rates to them. By combining block-based CS with saliency detection, we have implemented an adaptive sampling technique for CV tasks.

**Figure 3:**
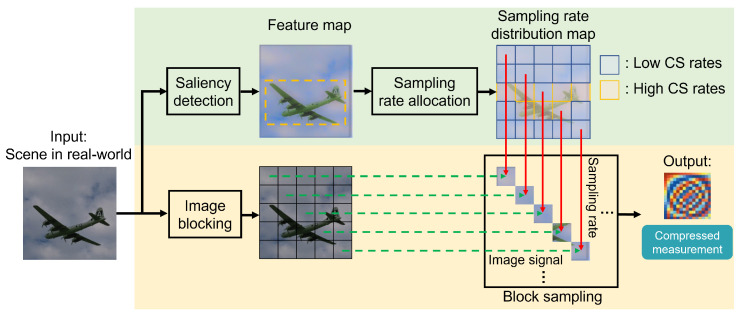
Structure of CV-oriented adaptive sampling [12]. Reproduced with permission from Luyang Liu, *Proceedings of the 5th ACM International Conference on Multimedia in Asia*; published by ACM, 2023.

The concept of the proposed sampling technique is illustrated in Figure 3. Here, the yellow part represents block sampling, while the green part represents the saliency-based sampling rate allocation. Based on block sampling, saliency detection is carried out for input signals. For saliency detection, we perform extraction on the input with a modified MobileNetV3 [25] for obtaining a feature distribution map. Based on the differences in feature weights between each block, we can discriminate between salient and non-salient blocks, and different sampling rates are allocated to them. Finally, each block of the original image signal (green dashed lines) is sampled at the sampling rate (red solid lines) of the corresponding block in the sampling rate distribution map, and the sampling results are combined into the compression measurement. More details about saliency detection are introduced below.

### 3.1. Saliency Detection

Considering the efficiency of processing on edge devices, we need to control the computational cost of the saliency detection part. In this work, we utilize lightweight MobileNetV3 [25] as the saliency feature extractor. MobilenetNetV3 is constructed based on depthwise separable convolution, and its feature extraction backbone contains only 0.47 M parameters and has only about 10 ms latency on edge devices [26]; these characteristics are much smaller than those of other current mainstream CNNs. MobilenetNetV3 fully meets the requirements of low cost and real-time operation, so there is no need to be concerned with a complexity increase associated with its introduction. The original MobileNetV3 uses a stepwise upsampling operation in the decoder to recover feature map specifications. Note that for the saliency detection part of this research, we only use the MobileNetV3 backbone to get the feature map rather than for subsequent predictions such as classification and segmentation. Therefore, the computational complexity expense in the decoder is completely unnecessary, and we simplified the structure of MobileNetV3 from the dimensional recovery phase.

The structure of the modified MobileNetV3 is shown in Table 1. Each bottleneck contains a 3 × 3 depthwise convolution and an SE attention layer. Specifically, in layer 17, we use one DUpsampling layer to replace the original decoder part in order to achieve fast, one-step recovery of the feature map to the same size as the original input. DUpsampling is supposed to be used for fine-grained recovery of target edges in semantic segmentation tasks, but it has also shown effectiveness in cross-dimensional feature map size recovery [27]. Compared to traditional bilinear upsampling, DUpsampling only applies 1 × 1 convolution to the spatial dimension and is based on the correlation between each pixel and rearranges channel vectors. This allows DUpsampling to recover from low-level dimensions to high-level dimensions in one step.

Meanwhile, without changing the backbone structure of MobileNetV3, it is still able to utilize the pre-training weights on the ImageNet dataset [28]. Compared to simple stacking of several convolutional layers, using the pre-trained feature extractor is more effective for determining saliency information from complex backgrounds [29]. As shown in Formula (1), for an *Input* whose length, width, and number of channels are *H*, *W*, and three, respectively, an output saliency feature map *S* with the same size as the *Input* but with one channel is obtained after convolution processing and dimension recovery by the CNN (modified MobileNetV3). Here, H×W×3 and H×W×1 represent the dimensions of the *Input* and *S*, respectively:

(1) $$\begin{array}{c} S_{\left(H\times W\times 1\right)}=CNN\left(Input_{\left(H\times W\times 3\right)}\right). \end{array}$$

### 3.2. Adaptive Sampling

In some previous works on discrete cosine transform (DCT)-based feature extraction, the researchers blocked the original image in order to calculate the DCT coefficient weights of each block and verified that the feature energy of the image is mainly concentrated in the block that has a higher-than-average DCT coefficient weight [30,31]. With reference to this fact, to correspond to subsequent block sampling, we block the feature map *S* obtained in the previous section and calculate the feature weight of each block to generate the block weight distribution map *W*. We determine the salient parts based on *W*. Assuming that the block size in block-based sampling is set to b×b, we divide pixels at every b×b position in the original feature map *S* into a block $S_{\left[b,b\right]}$ with kernel size b×b. Sum-pooling pools the summed values inside the scanning kernel [32]. While we can allocate finer-grained sampling rates for the blocks if we use smaller block sizes, the computational complexity increases with increasing the number of blocks. Let $W_{i,j}$ denote the feature value of the (*i, j*) block, and $S_{\left[b_{i},b_{j}\right]}$ represents a b×b-sized region corresponding to $W_{i,j}$ in the original feature map *S*. We use sum-pooling to accumulate the feature values of each pixel in the $S_{\left[b_{i},b_{j}\right]}$ block and obtain the feature weight of the block $W_{i,j}$, given by:

(2) $$\begin{array}{c} W_{i,j}=\sum_{i=1}^{W/b}\sum_{j=1}^{H/b}S_{\left[b_{i},b_{j}\right]}. \end{array}$$

The proposed technique allocates the sampling rates for each block from the feature differences. The blocks, which contain the potential interest for the CV task (e.g., semantic targets), are often accompanied by rich textures or distinct edges. Such blocks have high feature weights and can be considered salient blocks. The drawback of this proposal lies in that saliency detection could fail if textures and edges are unclear. Hence, pre-processing approaches, which are out of our scope in this paper, are sometimes required in advance.

Refer to [30,31]; our scheme is to calculate the average of the block weight distribution map *W* and use it as a criterion for determining salient blocks. The number of blocks and a cumulative $W_{i,j}$ for all blocks can derive the average feature value, which is represented as a threshold *t* in the following formula:

(3) $$\begin{array}{c} t=\frac{b^{2}\sum_{i=1}^{W/b}\sum_{j=1}^{H/b}W_{i,j}}{H\times W}. \end{array}$$

When a feature value on (*i, j*) is larger than the threshold *t*, the block at the current position is discriminated as a salient block and given a high sampling rate $r_{high}$; otherwise, a low sampling rate $r_{low}$ is given for the non-salient block. The threshold was pre-determined by the authors based on previous research. The formula is given below:

(4) $$\begin{array}{c} R_{i,j}=\left\{\begin{array}{c} \;\;r_{high},\;\;\mathrm{if}\;W_{i,j}>t\\ r_{low},\;\;\mathrm{otherwise} \end{array}\right. \end{array}$$

where $R_{i,j}$ represents the sampling rate value at the position of the *i*-th row and *j*-th column. In this way, the sampling rate distribution is generated. Finally, according to the sampling rate distribution map, each block is sampled at different sampling rates. With the aforementioned technique, we can discriminate between the salient and non-salient blocks of an image for sampling. Like other CS techniques, users can set the sampling rate according to their needs. In order to improve the inference accuracy for CV at low sampling rates, we want to retain as much information as possible that is useful for CV during the sampling process. Therefore, $r_{high}$ and $r_{low}$ have extremely different values, and the weight of useless information in the compressed measurements is reduced by setting a very low $r_{low}$. We tested a variety of combinations of $r_{high}$ and $r_{low}$; see Section 4 following for a detailed exploration of sampling rates.

**Figure 4:**
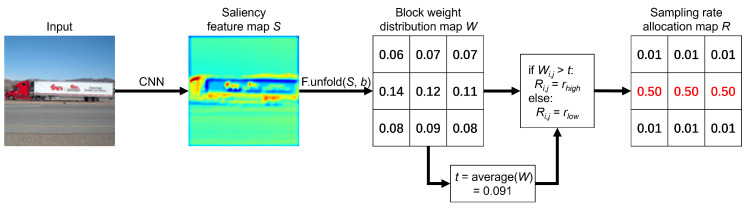
The process of adaptive sampling rate allocation: *b* represents the block size, $r_{high}$ and $r_{low}$ denote the high (red) and low (black) sampling rates, respectively, and $W_{i,j}$ and $R_{i,j}$ represent the value at the position of the *i*-th row and *j*-th column [12]. Reproduced with permission from Luyang Liu, *Proceedings of the 5th ACM International Conference on Multimedia in Asia*; published by ACM, 2023.

We illustrate the transformation from the input to the sampling rate distribution map that is implemented based on the proposed allocation scheme. For example, as shown in Figure 4, an input image of size 96 × 96 is extracted by the CNN mentioned in Section 3 to derive the saliency feature map *S*. Then, based on the pre-set block size of 32 × 32, the original image is divided into nine same-sized blocks, and the feature weights of each block are obtained. The average block weight is derived to be 0.091. Corresponding to the original image, it can be seen that the block in the middle row, where the truck is located, has a higher feature weight than the average weight and is allocated a high sampling rate (0.50). The rest of the background parts, i.e., the sky and the road, have feature weights that are lower than the mean average weight and are allocated a low sampling rate (0.01). Based on the above process, the transformation of the original input image to the sampling rate distribution map *R* is implemented. Finally, each block of the original image is sampled according to the corresponding sampling rates in *R*.

Regarding block sampling, here, we refer to the learned sampling matrix in the work of [33], which does not need to be transferred from the encoder to the decoder, thus eliminating the extra transmission cost. The sampling rate determines the size of the sampling matrix $\Phi \in R^{M\times N}$. Here, *N* is the number of columns of the sampling matrix, which corresponds to the dimension of the original input signal $x\in R^{N}$. *M* is the number of rows of the sampling matrix, which stands for the number of sampled measurements. Therefore, *M* is proportional to the sampling rate. For the original input, we unfold it into blocks of the same b×b size. The term k denotes the current row of Φ. The first row corresponds to the upper left block in the original input. Each block $x_{k}$ is sampled by its corresponding sampling matrix $\Phi_{r_{k}}$. The block compressed sampling can be expressed as:

(5) $$\begin{array}{c} y_{k}=\Phi_{r_{k}}x_{k} \end{array}$$

where $y_{k}$ is the result of compressed measurements. And $r_{k}$ is the corresponding allocated sampling rate in R.

## 4. Experiments

**Figure 5:**
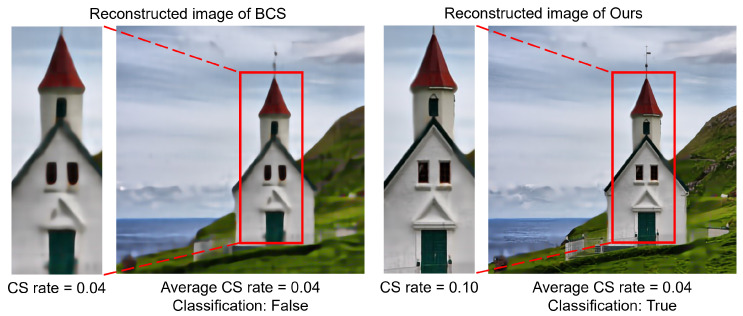
Classification error due to information loss in the reconstructed image. The left shows the image by uniform sampling (BCS) with incomplete reconstruction due to missing features of the original signal, which induces classification errors; the right shows the image by *Ours* with recognizable reconstruction due to adaptive sampling so that the feature information of the target is preserved.

**Figure 6:**
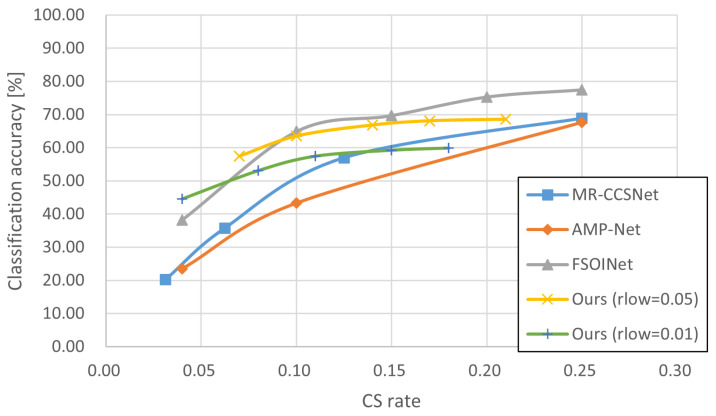
Comparison of results for state-of-the-art CS techniques on STL10 dataset [34].

**Figure 7:**
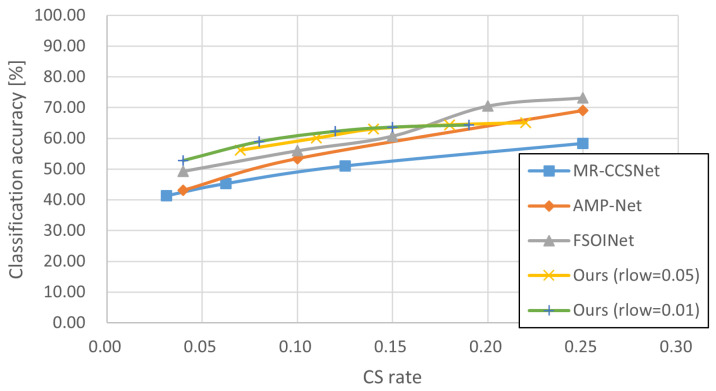
Comparison of results for state-of-the-art CS techniques on Intel dataset [35].

**Figure 8:**
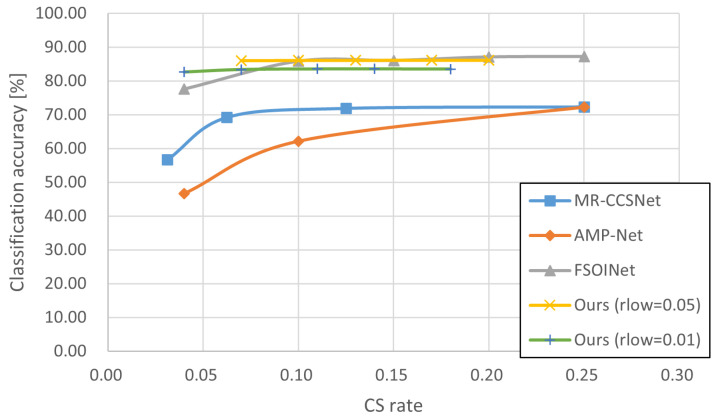
Comparison of results for state-of-the-art CS techniques on Imagenette dataset [36].

In this section, we conducted experiments in order to demonstrate the effectiveness of our proposed technique. The experiments tested and compared a variety of sampling techniques consisting of baseline sampling techniques with certain reconstruction techniques (Table 2, Table 3 and Table 4, and gives the example of reconstructed image in Figure 5) and state-of-the-art techniques (Figure 6, Figure 7 and Figure 8), and the accuracy of CV tasks is compared.

### 4.1. Classification

#### 4.1.1. Setup

To demonstrate the effectiveness of the proposal that improves the accuracy of the classification task, we conducted the following experiments. We first compared our proposed adaptive sampling technique with baseline techniques, namely BCS [8], BCS-PCT [9], and BCS-asymmetry [10]. BCS uses block-based uniform sampling, and BCS-PCT and BCS-asymmetry use adaptive sampling. BCS-PCT implements saliency detection and sampling rate allocation based on the pulsed cosine transform (PCT), while BCS-asymmetry considers the similarity between blocks to achieve asymmetric block segmentation and fine-grained sampling rate allocation. The above three techniques and the proposed technique are all based on block sampling implementation, and here, we compare sampling principles to confirm the effectiveness of the adaptive technique. Subsequently, the proposal will also be compared with state-of-the-art CS techniques proposed in recent years.

Regarding the reconstruction part of CS, we adopt a U-Net-based method from [37]. Much research on image restoration has shown that deep convolutional neural networks can effectively solve inverse problems in the image prior. In this work, we make the reconstruction network learn the mapping between the compressed measurements and the images in order to achieve the recovery of visualized results. Specifically, the structure of the reconstruction network is the backbone of the U-Net, which contains four scales. Each scale has a skip connection between upsampling and downsampling. Each upsampling and downsampling operation contains four residual blocks. Note that this work is concerned with the improvement of the sampling phase, and image recovery is not the focus at this time. The experiments here fixed the reconstruction part in order to compare the sampling methods fairly. Each sampling technique was combined with the reconstruction model component to form a complete CS network. For fair comparison and to verify the generalizability of the proposed technique, all CS networks were trained on the Berkeley Segmentation Dataset (BSD) [38], which contains 400 images cropped to 128 × 128 patch sizes. Each network was implemented with PyTorch, used 200 training epochs on an NVIDIA RTX 3070 GPU, employed the Adam optimizer, and had the learning rate set to 0.0001. For the proposed techniques, we adopted a pre-trained MobileNetV3 for saliency detection. The testing scenario was as follows: we were given the STL10 dataset [34], Intel image classification dataset [35], and Imagenette dataset [36] with image sizes of 96 × 96, 150 × 150, and 512 × 512, respectively, as input.

We prepared ten scenarios using different sampling rate combinations that varied from 0.05 to 0.01 for non-salient blocks (hereafter called $r_{low}$) and from 0.50 to 0.10 for salient blocks (hereafter called $r_{high}$). We employed average sampling rates in order to fairly compare the other sampling techniques since our proposal allocates different sampling rates for blocks, and the block size was set to 8 × 8. In addition, we also evaluated classification accuracy for different block sizes. Our proposed technique was compared to BCS, where the block sizes were set to 32 × 32, 16 × 16, and 8 × 8. The sampling rate allocation was consistent with the above for a total of ten scenarios. All CS techniques were evaluated in terms of the average CS rate (sampling rate), reconstructed image quality, and classification accuracy, and this paper specifically focuses on the performance of classification accuracy. As a reference, we also give the classification accuracies of the uncompressed original dataset at the top of Table 2, Table 3 and Table 4. The image quality of the reconstructed images using each CS technique was evaluated in terms of the PSNR (peak signal-to-noise ratio) and SSIM (structural similarity), which indicate the similarity of a reconstructed image to an original image. Classification accuracy was evaluated based on the three popular neural networks for classification: Xception [39], ResNet152 (hereafter called ResNet) [40], and DenseNet201 (hereafter called DenseNet) [41]. Each network was implemented with PyTorch and was trained for 200 epochs on an NVIDIA RTX 3070 GPU at a learning rate of 0.1 on the STL10, Intel, or Imagenette dataset.

Next, we compared the proposed technique with the state-of-the-art CS techniques: MR-CCSNet, which collects global information through multiple measurements and uses it for high-quality image reconstruction [15]; AMP-Net, which constructs a deep network based on an iterative denoising process to remove blurring from reconstructed images [42]; and FSOINet, which trains and strengthens the sampling matrix by learning the mapping of the original signal in the pixel space in relation to the feature space [43]. The other experimental setup was similar to the aforementioned content: we trained MR-CCSNet, AMP-Net, and FSOINet for 200 epochs on the BSD500 dataset [44], which is commonly used for CS training. To compare them, we used the proposed sampling technique whereby 0.05 and 0.01 were used for non-salient blocks as $r_{low}$ and 0.50 to 0.10 were used for salient blocks as $r_{high}$. In the experiments, we employed the different sampling rates for each of the three CS techniques (MR-CCSNet, with sampling rates of 0.03125, 0.06250, 0.12500, and 0.25000; AMP-Net, with sampling rates of 0.04000, 0.10000, and 0.25000; and FSOINet, with sampling rates of 0.04000, 0.100000, 0.15000, 0.20000, and 0.25000). The state-of-the-art CS techniques faced the limitations of specific parameters of the sampling matrix and of adopting distinct strategies for reconstructing images at different sampling rates. It should be noted that we can thus hardly compare them in fairness since these techniques can only achieve sensing at specific sampling rates. While it is challenging to conduct a perfectly fair comparison due to the non-uniformity of the sampling rate, it is still feasible to evaluate the effectiveness of each CS technique at image classification tasks by observing the curve’s height and direction in the graph. The results are represented by polylines because inference accuracy generally shows a positive correlation with the sampling rate. We expect this work to address the problem of reduced accuracy of reconstructed images under scenarios of low sampling rates, with the objective of decreasing the amount of data transmitted. Consistent with previous experiments, all CS networks were trained on BSD with the same parameter settings. The trained networks were given the STL10 dataset, Intel image classification dataset and Imagenette dataset, and the reconstructed images obtained by the state-of-the-art CS techniques were inferred by the classification networks, which are the same as the previous experiment: Xception [39], ResNet [40], and DenseNet [41] trained with the same parameter settings.

#### 4.1.2. Results

Table 2, Table 3 and Table 4 display the experimental results for the STL10, Intel, and Imagenette datasets, respectively, and compare four CS sampling techniques at ten different average sampling rates. The results include two metrics: image quality, evaluated by the averages of the PSNR and SSIM, and classification accuracy, which is compared at ten sampling rates using four classification models. For example, in Table 2, at the average CS rate of 0.21, the proposed technique *Ours* employed two sampling rates, with $r_{low}$ set to 0.05 and $r_{high}$ to 0.50, resulting in an overall average CS rate of 0.21. Other CS techniques are also fixed at 0.21 for fair comparison (i.e., having the same average sampling rate). The subsequent columns show the reconstructed image quality and classification accuracy of each technique. Here, “Difference” shows the differences in the average classification accuracies between the proposed technique and other techniques. The “-” means that the proposed technique is better than the other techniques.

Table 2 and Table 3 demonstrate that our proposal generally outperforms the other techniques in terms of classification accuracy, except for the case using an average sampling rate of 0.18 in Table 2. The results indicate that *Ours* is generally superior to the others in terms of classification accuracy because blocks that are identified as salient are allocated a higher sampling rate. Compared to BCS, *Ours* achieves higher classification accuracy by up to 26.23% in the case of 0.10 in Table 2 and up to 11.69% in the case of 0.15 in Table 3 for each champion case. Although BCS-PCT and BCS-asymmetry are superior to BCS, they cannot exceed *Ours* except for the 0.18 case in Table 2. Especially when the average sampling rate decreases, *Ours* shows the tendency to achieve higher classification accuracy. In terms of PSNR and SSIM, there are also quite a few cases for which *Ours* have higher values than the other techniques, as shown for sampling rates of 0.21, 0.17, 0.14, 0.10, 0.07, and 0.04 in Table 2 and 0.22, 0.18, 0.14, 0.11, 0.08, 0.07, and 0.04 in Table 3, respectively. Although the compared techniques aim for high image quality, *Ours* still shows an advantage in terms of image quality over the other techniques due to partly allocating high sampling rates to salient blocks, but the image quality does not always synchronize with the classification accuracy since the image quality in *Ours* largely depends on the size of the object being classified. The larger the target, the higher the number of salient blocks as a proportion of the whole image. There are the cases where *Ours* shows lower PSNRs and SSIMs compared to the others, as shown for sampling rates of 0.18, 0.15, 0.11, and 0.08 in Table 2 and 0.19, 0.15, and 0.12 in Table 3, respectively, but we achieve higher accuracy than the others since *Ours* is not oriented to the image quality but to the classification accuracy. Figure 5 exemplifies the visual differences between BCS and *Ours* for the 0.04 sampling rate. The original image is labeled “church”. In the reconstructed image from the uniform CS (BCS) on the left, the target church becomes blurred at the low sampling rate (0.04), leading to a classification error. In contrast, the reconstructed image from our proposed technique on the right side shows the target church with comparative clarity, leading to correct classification by focusing on the salient target using a high sampling rate (0.10). By sampling the remaining non-salient blocks (sky and greenfield) at a lower sampling rate (0.01), we reduced the weighting of non-essential information, thus decreasing the overall sampling cost. Despite having lower image qualities, *Ours* achieved higher classification accuracy in most cases, except for the 0.18 case. This implies that the overall image quality does not always contribute to high classification accuracy. However, in the 0.18 case, there is a possibility that feature extraction in *Ours* might fail, leading to higher sampling rates being allocated to non-salient blocks. This case leaves a future challenge, which is to detect true saliency maps. In addition, we still need to note that on both the STL10 and Intel datasets, there is a significant loss of accuracy in the compressed-sampled image compared to the original image due to the unavoidable loss of information at very low sampling rates. How to reasonably set the sampling rates allocated to the salient and non-salient blocks in order to maximize the accuracy preservation is still a major work for the future.

Table 4 presents the results for the Imagenette dataset, which contains larger, 512 × 512 images compared to the STL10 and Intel datasets. While *Ours* demonstrates advantages in classification accuracy for sampling rates lower than 0.10, in other cases, it appears to be inferior to the other techniques. One reason behind these results is that the classification task for the Imagenette dataset might be too easy to show significant differences among the techniques, given the rich information present in the original images. As seen from the table, the classification accuracies are consistently high for all techniques and are quite close to each other, making it challenging to compare them effectively. Even when the rich information is significantly reduced by compression, the classification accuracy remains high for all four classification models. However, *Ours* maintains relatively high accuracy at 82.65%—close to that of the original image and an improvement of up to 18.25% over the other techniques—at a sampling rate of 0.04. This suggests that our proposed technique could be particularly useful for sampling images with originally poor information that were captured using reasonable cameras equipped on edge devices with limited resources.

Table 5 explores the results using different block sizes for sampling. In the headers, we need to note that the average CS rate represents the average of the average CS rates in Table 2, Table 3 and Table 4 for *Ours* and BCS using three different block sizes. Overall, for a block size of 8 × 8, *Ours* shows the highest classification accuracy since it can allocate sampling rates for finer-grained blocks in this case versus for the other block sizes. Unlike *Ours*, BCS has almost the same accuracy for different block sizes due to its uniform sampling. Compared to BCS, *Ours* outperforms 16.24% for STL10 and 10.18% for Intel. However, there is a marginal difference for the Imagenette dataset, where both techniques show close accuracy regardless of block sizes.

As described in Section 4.1.1, some SOTA techniques can only be implemented at specified sampling rates. These inherent limitations make it difficult to compare them at the same sampling rate. Therefore, some curve graphs of sampling rate classification accuracy are used here instead of tables to visually show the performance differences of each CS technique. Figure 6, Figure 7 and Figure 8 display the experimental results for the STL10, Intel, and Imagenette datasets, respectively, to compare our proposal with three state-of-the-art CS techniques at different sampling rates. Overall, the results show a positive correlation between the inference accuracy and sampling rate.

The results in Figure 6, Figure 7 and Figure 8 show that the polylines of *Ours* are higher than those of MR-CCSNet and AMP-Net when the sampling rate is less than 0.20, which demonstrates that the proposal performs better than MR-CCSNet and AMP-Net at low sampling intervals for the image classification task. Although FSOINet initially performed well due to its superior image reconstruction capabilities, it inevitably experienced a loss in accuracy as the sampling rate decreased and the amount of data available for reconstruction was reduced. The performance curve of FSOINet shows a sharp decline and is eventually overtaken by our proposed technique. Specifically, Figure 8 illustrates that in scenarios with large-sized images and complex backgrounds, while the performance curves of other CS techniques drop sharply, the curve of our proposal remains stable. Even at very low sampling rates, it maintains a high level of precision, comparable to the original image. This demonstrates the effectiveness of our proposed scheme for reducing the amount of data required for downstream classification tasks, offering a viable solution to lower the transmission costs for edge devices.

Furthermore, we note that for our proposal, when the sampling rate ($r_{low}$) of the non-salient blocks is set to 0.01, it consistently exhibits inferior classification accuracy compared to instances where the sampling rate is set to 0.05. This discrepancy suggests that non-salient blocks, despite lacking information about predefined classification targets, may contribute to target boundary determination and subsequently impact final classification results. Therefore, future work is to find the best combination of non-salient and salient block sampling rates to achieve the highest accuracy.

### 4.2. Object Detection

Image classification requires classifying an entire image into predefined categories or classes, such as identifying whether an image contains a cat or a dog [45,46]. Object detection requires identifying and locating objects of interest within an image, such as detecting and locating multiple faces in a crowd [47,48]. Compared to image classification, the object detection task is more complex and difficult; the network has to understand both global and local features of the image to localize the distribution of objects [49]. Therefore, the amount of information required to implement an image classification task is usually less than the amount of information required to implement an object detection task [50]. To verify the effectiveness of the proposal at improving the detection accuracy, we conducted the following experiments.

#### 4.2.1. Setup

The experimental scenario is as follows: we are given the KITTI 2D object detection dataset [51] as input. The KITTI dataset is currently the main road object detection dataset for autonomous driving and has an input size of 1224 × 370. Using the KITTI dataset for experimental testing can simulate the scenario of the proposed technique in real IoT and embedded CV applications. The experimental details here are consistent with those in Section 4.1.1.

We first compared the four baseline sampling techniques: BCS, BCS-PCT, BCS-asymmetry, and *Ours*. The settings for training, sampling rate combinations, and block sizes were the same as those listed in Section 4.1.1. The compressed measurements obtained by sampling compression were reconstructed by the same method and fed into the object detection network for accuracy testing. For detection, we utilzied YOLOv3 [52], which has been well-established by other works such as [53,54,55,56] for inference using the KITTI dataset.

YOLOv3 was implemented with PyTorch and was trained for 100 epochs on an NVIDIA RTX 3070 GPU; we employed the Adam optimizer with a learning rate of 0.01. All CS sampling techniques were evaluated in terms of the average CS rate (sampling rate), reconstructed image quality, and detection accuracy, and this part specifically focused on detection accuracy performance. The evaluation metrics for detection accuracy include precision, recall, F1-score, and mAP.

Similar to in Section 4.1, we evaluated the effectiveness of our proposal compared to state-of-the-art CS techniques (MR-CCSNet, AMP-Net, and FSOINet) within the context of object detection tasks. The experimental setup and model training closely mirrored those outlined in Section, with the trained models tested on the KITTI dataset. The evaluation of detection accuracy was conducted through object detection using YOLOv3 on the reconstructed images generated by each CS technique, with a specific emphasis on the mAP.

#### 4.2.2. Results

Table 6 presents the results on the KITTI dataset. The results are discussed in two parts according to the sampling rate interval.

For the high sampling rate interval from 0.20 to 0.11, *Ours* outperforms the other CS sampling techniques in terms of reconstructed image quality and detection accuracy when the sampling rate allocated to the non-salient blocks $r_{low}$ is 0.05. When the sampling rate $r_{low}$ is 0.01, *Ours* generally outperforms BCS. There were some cases that were slightly worse than for the other two adaptive CS sampling techniques in terms of image quality and detection accuracy (usually by only 0.1). However, the differences seem negligible.

**Figure 9:**
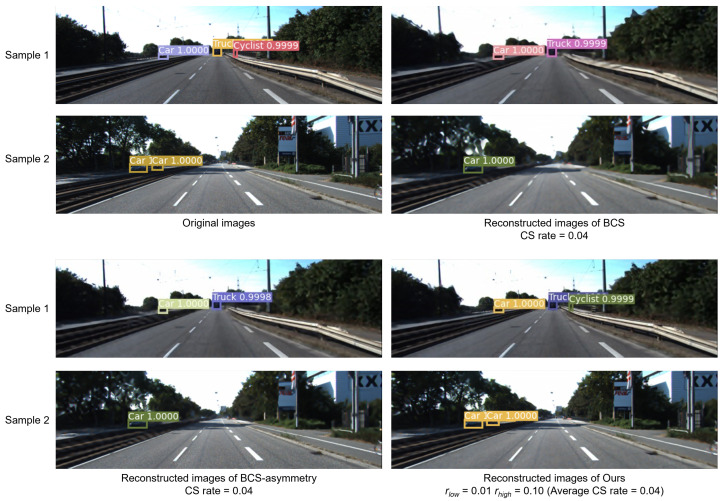
Object detection results of reconstructed images with different sampling techniques.

For the low sampling rate interval from 0.10 to 0.04, *Ours* outperforms the other CS sampling techniques in terms of detection accuracy. In particular, when the average sampling rates are 0.07 and 0.04, *Ours* achieves higher detection accuracy even though it is lower in reconstructed image quality than BCS-asymmetry, which is specifically designed to enhance visualization. This also shows that high inference accuracy for CV tasks does not depend entirely on high image quality. When the average sampling rate is 0.04, the mAP of 0.387 for BCS drops to a little more than half of that of the original image, and the two existing adaptive CS sampling techniques, BCS-PCT and BCS-asymmetry, also have significant decreases in detection accuracy, but *Ours* still achieves a higher mAP of 0.723: maintaining a similar level of accuracy as the original image. Figure 9 shows the object detection results for the original image and the reconstructed images of BCS, BCS-asymmetry, and *Ours* (the average sampling rate is 0.04). It can be seen that at lower sampling rates, there is significant mis-detection in the reconstructed images of BCS and BCS-asymmetry. In contrast, at the same sampling rate, the reconstructed images of *Ours* still correctly portray predefined objects that cannot be recognized in the images from BCS and BCS-asymmetry, such as the cyclist in Sample 1 and the car in Sample 2 from Figure 9. In contrast, the results of *Ours* show that the detection of each predefined object is achieved with confidence that is very close to that of the original image. Although the pixel size of the predefined object is very small relative to the whole image, its features are transmitted through our proposal sampling technique. This is attributed to the fact that by allocating a higher sampling rate to the saliency parts in the original image, the information required for the object detection task is preserved. This also proves that the proposed technique can still achieve high accuracy with a lower sampling rate and higher degree of data compression for the object detection task.

**Figure 10:**
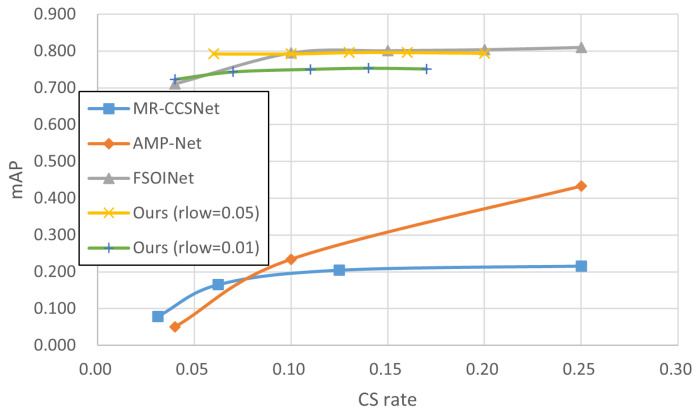
Comparison of results for state-of-the-art CS techniques on KITTI dataset [51].

To comprehensively verify the effectiveness of our proposal, we compared it with the most advanced CS techniques. Figure 10 presents a graphical representation of curves depicting the relationship between the detection mAP and sampling rate for various CS techniques on the KITTI dataset. An analysis of Figure 10 reveals distinctive performance characteristics. Our proposal consistently outperforms MR-CCSNet and AMP-Net in terms of detection accuracy, showcasing superior performance across varying sampling rates. Notably, our proposal demonstrates detection accuracy comparable to that of FSOINet when the sampling rate exceeds 0.10 (when $r_{low}$ of *Ours* is 0.05). As the sampling rate decreases, FSOINet exhibits a decline in detection accuracy: this is particularly noticeable when the sampling rate is lower than 0.10. In contrast, our proposal maintains a high level of detection accuracy even under conditions of very low sampling rates. This result underscores the robustness of our proposal and its versatility for effectively addressing a broad spectrum of CV tasks.

## 5. Conclusions and Future Remarks

This paper presents an adaptive sampling technique in CS with the aim of improving the accuracy of CV tasks even at low sampling rates. Our contribution to the field of sensing technology lies in providing a framework that enables sensor systems to intelligently prioritize and transmit the most relevant data. Our technique ensures that essential information required for CV tasks is retained, thereby improving the overall efficacy of sensor-based data acquisition systems. The experimental results validate that our technique yields superior classification and object detection accuracy on various datasets collected by real sensor devices. This highlights the potential of our technique to maintain high inference performance while significantly reducing data transmission costs in sensor-rich but bandwidth-constrained environments. Future work will be to find the best combination of non-salient and salient block sampling rates to achieve the highest accuracy and to seek better salient block detection.

## Figures and Tables

**Table 1:** Network layer structure of one-step recovery MobileNetV3.

Layer	Input	Operator	Output Channel	Stride
1	H × W × 3	conv2d	16	2
2	(H × W)/4 × 16	bottleneck, 3 × 3	16	1
3	(H × W)/4 × 16	bottleneck, 3 × 3	24	2
4	(H × W)/16 × 24	bottleneck, 3 × 3	24	1
5	(H × W)/16 × 24	bottleneck, 5 × 5	40	2
6	(H × W)/64 × 40	bottleneck, 5 × 5	40	1
7	(H × W)/64 × 40	bottleneck, 5 × 5	40	1
8	(H × W)/64 × 40	bottleneck, 3 × 3	80	2
9	(H × W)/256 × 80	bottleneck, 3 × 3	80	1
10	(H × W)/256 × 80	bottleneck, 3 × 3	80	1
11	(H × W)/256 × 80	bottleneck, 3 × 3	80	1
12	(H × W)/256 × 80	bottleneck, 3 × 3	112	1
13	(H × W)/256 × 112	bottleneck, 3 × 3	112	1
14	(H × W)/256 × 112	bottleneck, 5 × 5	160	2
15	(H × W)/1024 × 160	bottleneck, 5 × 5	160	1
16	(H × W)/1024 × 160	bottleneck, 5 × 5	160	1
17	(H × W)/1024 × 160	DUpsampling	1	1
Output	H × W × 1	-	-	-

**Table 2:** Comparison of results [12] for BCS [8], BCS-PCT [9], and BCS-asymmetry [10] on STL10 dataset [34]. Reproduced with permission from Luyang Liu, *Proceedings of the 5th ACM International Conference on Multimedia in Asia*; published by ACM, 2023.

Average CS Rate	Sampling Technique	CS Rate			Reconstructed Image Quality		Classification Accuracy [%]				
		**Saliency ($r_{\mathrm{high}}$)**	**Non-Saliency ($r_{\mathrm{low}}$)**		**PSNR [dB]**	**SSIM**	**Xception**	**ResNet**	**DenseNet**	**Average**	**Difference**
1.00	Original image		1.00		-	-	86.32	80.76	76.71	81.26	-
0.21	BCS		0.21		24.33	0.76	54.48	55.17	51.45	53.70	−14.88
	BCS-PCT		0.21		25.58	0.81	62.42	62.17	57.97	60.85	−7.73
	BCS-asymmetry		0.21		26.20	0.83	66.90	65.13	60.80	64.28	−4.30
	Ours	0.50		0.05	**26.40**	**0.85**	**71.91**	**69.33**	**64.50**	**68.58**	±0.00
0.18	BCS		0.18		24.33	0.76	54.48	55.17	51.45	53.70	−6.20
	BCS-PCT		0.18		25.42	0.80	61.20	61.53	57.08	59.94	+0.03
	BCS-asymmetry		0.18		**26.03**	**0.83**	**65.91**	**64.55**	**60.11**	**63.52**	+3.62
	Ours	0.50		0.01	22.97	0.73	63.42	59.23	57.06	59.90	±0.00
0.17	BCS		0.17		24.33	0.76	54.48	55.17	51.45	53.70	−14.42
	BCS-PCT		0.17		25.37	0.80	60.78	61.31	56.90	59.66	−8.46
	BCS-asymmetry		0.17		25.97	0.82	65.35	64.23	59.91	63.16	−4.96
	Ours	0.40		0.05	**26.27**	**0.85**	**71.16**	**69.05**	**64.16**	**68.12**	±0.00
0.15	BCS		0.15		23.35	0.71	46.60	45.67	44.91	45.73	−13.52
	BCS-PCT		0.15		24.45	0.76	55.87	55.32	52.45	54.55	−4.70
	BCS-asymmetry		0.15		**25.04**	**0.79**	61.21	**59.52**	56.31	59.01	-0.23
	Ours	0.40		0.01	22.89	0.72	**62.51**	58.62	**56.60**	**59.24**	±0.00
0.14	BCS		0.14		23.35	0.71	46.60	45.67	44.91	45.73	−21.11
	BCS-PCT		0.14		24.38	0.76	55.25	54.93	52.02	54.07	−12.77
	BCS-asymmetry		0.14		24.97	0.79	60.62	59.02	56.01	58.55	−8.29
	Ours	0.30		0.05	**26.06**	**0.84**	**69.41**	**68.05**	**63.05**	**66.84**	±0.00
0.11	BCS		0.11		23.35	0.71	46.60	45.67	44.91	45.73	−11.71
	BCS-PCT		0.11		24.16	0.75	53.57	53.12	50.56	52.42	−5.02
	BCS-asymmetry		0.11		**24.72**	**0.77**	58.48	57.15	54.22	56.62	−0.82
	Ours	0.30		0.01	22.77	0.71	**60.05**	**57.16**	**55.11**	**57.44**	±0.00
0.10	BCS		0.10		22.14	0.64	38.78	35.27	37.81	37.29	−26.23
	BCS-PCT		0.10		23.11	0.70	48.37	46.11	46.78	47.09	−16.43
	BCS-asymmetry		0.10		23.68	0.73	54.28	51.88	51.35	52.50	−11.02
	Ours	0.20		0.05	**25.69**	**0.82**	**64.57**	**65.68**	**60.31**	**63.52**	±0.00
0.08	BCS		0.08		22.14	0.64	38.78	35.27	37.81	37.29	−15.80
	BCS-PCT		0.08		22.92	0.69	46.43	43.72	44.72	44.96	−8.13
	BCS-asymmetry		0.08		**23.46**	**0.72**	52.10	49.37	49.31	50.26	-2.83
	Ours	0.20		0.01	22.56	0.69	**54.30**	**54.02**	**50.95**	**53.09**	±0.00
0.07	BCS		0.07		22.14	0.64	38.78	35.27	37.81	37.29	−20.15
	BCS-PCT		0.07		22.81	0.68	44.71	42.43	43.65	43.60	−13.84
	BCS-asymmetry		0.07		23.33	0.71	50.60	48.17	48.40	49.06	−8.38
	Ours	0.10		0.05	**24.99**	**0.79**	**56.90**	**60.07**	**55.33**	**57.43**	±0.00
0.04	BCS		0.04		19.99	0.50	28.88	22.06	27.66	26.20	−18.35
	BCS-PCT		0.04		20.58	0.54	32.63	26.75	31.47	30.28	−14.26
	BCS-asymmetry		0.04		21.08	0.58	37.63	32.82	36.07	35.51	−9.04
	Ours	0.10		0.01	**22.10**	**0.66**	**44.93**	**45.20**	**43.51**	**44.55**	±0.00

**Table 3:** Comparison of results for BCS [8], BCS-PCT [9], and BCS-asymmetry [10] on Intel dataset [35].

Average CS Rate	Sampling Technique	CS Rate			Reconstructed Image Quality		Classification Accuracy [%]				
		**Saliency ($r_{\mathrm{high}}$)**	**Non-Saliency ($r_{\mathrm{low}}$)**		**PSNR [dB]**	**SSIM**	**Xception**	**ResNet**	**DenseNet**	**Average**	**Difference**
1.00	Original image		1.00		-	-	88.56	87.13	68.66	81.45	-
0.22	BCS		0.22		24.39	0.73	53.20	48.96	67.53	56.56	−8.50
	BCS-PCT		0.22		25.20	0.77	56.06	53.86	67.53	59.15	−5.91
	BCS-asymmetry		0.22		25.70	0.79	58.26	56.03	67.50	60.60	−4.47
	Ours	0.50		0.05	**26.18**	**0.82**	**64.56**	**62.73**	**67.90**	**65.06**	±0.00
0.19	BCS		0.19		23.87	0.70	50.56	46.46	67.53	54.85	−9.49
	BCS-PCT		0.19		24.70	0.74	54.20	51.86	67.53	57.86	−6.48
	BCS-asymmetry		0.19		**25.17**	**0.76**	56.66	54.53	**67.66**	59.62	−4.73
	Ours	0.50		0.01	24.10	0.72	**62.53**	**63.70**	66.80	**64.34**	±0.00
0.18	BCS		0.18		23.87	0.70	50.56	46.46	67.53	54.85	−9.54
	BCS-PCT		0.18		24.66	0.74	54.06	51.63	67.46	57.72	−6.68
	BCS-asymmetry		0.18		25.12	0.76	56.76	54.30	67.66	59.57	−4.82
	Ours		0.40	0.05	**25.98**	**0.81**	**63.66**	**61.66**	**67.86**	**64.39**	±0.00
0.15	BCS		0.15		23.21	0.65	46.76	42.13	67.03	51.97	−11.69
	BCS-PCT		0.15		23.99	0.70	50.86	47.76	**67.16**	55.26	−8.40
	BCS-asymmetry		0.15		**24.43**	**0.73**	52.80	51.50	**67.16**	57.15	-6.51
	Ours	0.40		0.01	23.96	0.71	**61.93**	**62.40**	66.66	**63.66**	±0.00
0.14	BCS		0.14		23.21	0.65	46.76	42.13	67.03	51.97	−11.02
	BCS-PCT		0.14		23.94	0.70	50.66	47.73	67.20	55.20	−7.80
	BCS-asymmetry		0.14		24.38	0.72	53.03	51.06	67.20	57.10	−5.90
	Ours	0.30		0.05	**25.69**	**0.80**	**61.90**	**59.26**	**67.83**	**63.00**	±0.00
0.12	BCS		0.12		23.21	0.65	46.76	42.13	67.03	51.97	−10.33
	BCS-PCT		0.12		23.84	0.69	50.30	46.60	67.13	54.68	−7.62
	BCS-asymmetry		0.12		**24.26**	**0.72**	52.00	50.13	**67.33**	56.49	−5.81
	Ours	0.30		0.01	23.76	0.70	**59.70**	**60.60**	66.60	**62.30**	±0.00
0.11	BCS		0.11		23.21	0.65	46.76	42.13	67.03	51.97	−8.08
	BCS-PCT		0.11		23.79	0.69	50.20	46.23	67.30	54.58	−5.47
	BCS-asymmetry		0.11		24.19	0.71	51.70	49.40	67.33	56.14	−3.91
	Ours	0.20		0.05	**25.23**	**0.77**	**57.86**	**54.56**	**67.73**	**60.05**	±0.00
0.08	BCS		0.08		22.40	0.59	42.56	37.90	67.06	49.17	−9.81
	BCS-PCT		0.08		22.96	0.63	46.03	43.10	67.13	52.09	−6.90
	BCS-asymmetry		0.08		23.35	0.66	47.50	46.46	**67.43**	53.80	−5.19
	Ours	0.20		0.01	**23.43**	**0.67**	**55.06**	**55.16**	66.73	**58.98**	±0.00
0.07	BCS		0.07		22.40	0.59	42.56	37.90	67.06	49.17	−6.94
	BCS-PCT		0.07		22.88	0.63	45.40	42.63	67.30	51.78	−4.34
	BCS-asymmetry		0.07		23.26	0.65	47.53	45.66	67.30	53.50	−2.62
	Ours	0.10		0.05	**24.50**	**0.74**	**51.96**	**48.56**	**67.83**	**56.12**	±0.00
0.04	BCS		0.04		21.03	0.48	33.50	26.30	66.53	42.11	−10.73
	BCS-PCT		0.04		21.49	0.52	37.66	30.60	66.90	45.05	−7.79
	BCS-asymmetry		0.04		21.86	0.55	40.53	34.90	**67.16**	47.53	−5.31
	Ours	0.10		0.01	**22.88**	**0.64**	**46.93**	**44.66**	66.93	**52.84**	±0.00

**Table 4:** Comparison of results [12] for BCS [8], BCS-PCT [9], and BCS-asymmetry [10] on Imagenette dataset [36]. Reproduced with permission from Luyang Liu, *Proceedings of the 5th ACM International Conference on Multimedia in Asia*; published by ACM, 2023.

Average CS Rate	Sampling Technique	CS Rate			Reconstructed Image Quality		Classification Accuracy [%]				
		**Saliency ($r_{\mathrm{high}}$)**	**Non-SALIENCY ($r_{\mathrm{low}}$)**		**PSNR [dB]**	**SSIM**	**Xception**	**ResNet**	**DenseNet**	**Average**	**Difference**
1.00	Original image		1.00		-	-	90.03	85.40	84.86	86.76	-
0.20	BCS		0.20		29.43	0.85	88.64	83.01	84.06	85.24	−0.88
	BCS-PCT		0.20		30.79	0.88	88.98	83.94	84.51	85.81	−0.31
	BCS-asymmetry		0.20		31.37	0.89	89.01	84.23	**84.60**	85.95	−0.17
	Ours	0.50		0.05	**33.50**	**0.91**	**89.32**	**84.49**	84.54	**86.12**	±0.00
0.18	BCS		0.18		29.43	0.85	88.64	83.01	84.06	85.24	+1.74
	BCS-PCT		0.18		30.69	0.87	89.01	83.86	84.51	85.79	+2.29
	BCS-asymmetry		0.18		**31.27**	**0.88**	**89.12**	**84.12**	**84.66**	**85.97**	+2.47
	Ours	0.50		0.01	29.92	0.84	88.50	80.76	81.24	83.50	±0.00
0.17	BCS		0.17		29.43	0.85	88.64	83.01	84.06	85.24	−0.92
	BCS-PCT		0.17		30.63	0.87	88.98	83.86	84.54	85.79	−0.36
	BCS-asymmetry		0.17		31.21	0.88	89.07	84.09	84.71	85.96	−0.20
	Ours	0.40		0.05	**33.21**	**0.91**	**89.44**	**84.51**	84.51	**86.15**	±0.00
0.14	BCS		0.14		28.38	0.82	87.10	80.90	83.15	83.72	+0.18
	BCS-PCT		0.14		29.58	0.85	88.10	82.66	83.97	84.91	+1.37
	BCS-asymmetry		0.14		**30.15**	**0.86**	88.21	**83.29**	**84.09**	**85.20**	+1.66
	Ours	0.40		0.01	29.76	0.84	**88.44**	80.76	81.41	83.54	±0.00
0.13	BCS		0.13		28.38	0.82	87.10	80.90	83.15	83.72	−2.38
	BCS-PCT		0.13		29.51	0.85	87.93	82.81	84.03	84.92	−1.18
	BCS-asymmetry		0.13		30.07	0.86	88.21	83.15	84.12	85.16	−0.94
	Ours	0.30		0.05	**32.80**	**0.90**	**89.35**	**84.46**	**84.49**	**86.10**	±0.00
0.11	BCS		0.11		28.38	0.82	87.10	80.90	83.15	83.72	+0.19
	BCS-PCT		0.11		29.35	0.84	87.84	82.52	83.94	84.77	+1.24
	BCS-asymmetry		0.11		**29.91**	**0.85**	88.19	**83.01**	**84.12**	**85.11**	+1.58
	Ours	0.30		0.01	29.53	0.83	**88.44**	80.67	81.47	83.53	±0.00
0.10	BCS		0.10		27.02	0.77	84.51	75.61	80.16	80.09	−5.99
	BCS-PCT		0.10		28.17	0.81	86.39	79.68	82.15	82.74	−3.34
	BCS-asymmetry		0.10		28.72	0.82	86.71	80.36	82.69	83.25	−2.83
	Ours	0.20		0.05	**32.08**	**0.89**	**89.27**	**84.46**	**84.51**	**86.08**	±0.00
0.07	BCS		0.07		27.02	0.77	84.51	75.61	80.16	80.09	−3.28
	BCS-PCT		0.07		27.85	0.80	86.14	78.45	81.78	82.12	−1.25
	BCS-asymmetry		0.07		28.38	0.81	86.62	79.93	**82.15**	82.90	−0.47
	Ours	0.20		0.01	**29.11**	**0.82**	**88.21**	**80.53**	81.38	**83.37**	±0.00
0.07	BCS		0.07		27.02	0.77	84.51	75.61	80.16	80.09	−5.92
	BCS-PCT		0.07		27.85	0.80	86.14	78.45	81.78	82.12	−3.89
	BCS-asymmetry		0.07		28.38	0.81	86.62	79.93	82.15	82.90	−3.11
	Ours	0.10		0.05	**30.76**	**0.87**	**89.21**	**84.23**	**84.60**	**86.01**	±0.00
0.04	BCS		0.04		24.75	0.68	74.61	59.27	59.33	64.40	−18.25
	BCS-PCT		0.04		25.53	0.72	78.77	65.59	68.41	70.92	−11.73
	BCS-asymmetry		0.04		26.05	0.74	80.73	68.83	71.99	73.85	−8.80
	Ours	0.10		0.01	**28.26**	**0.80**	**87.47**	**79.53**	**80.96**	**82.65**	±0.00

**Table 5:** Comparison using three different block sizes.

Dataset	Block Size	Average CS Rate	Average Accuracy of BCS [%]	Average Accuracy of Ours [%]	Improvement in Accuracy of Ours Compared with BCS [%]
STL10 (96 × 96)	32 × 32	0.15	44.75	54.44	+9.69
	16 × 16	0.14	43.64	56.16	+12.52
	8 × 8	**0.13**	43.63	**59.87**	**+16.24**
Intel (150 × 150)	32 × 32	0.14	49.61	59.21	+9.60
	16 × 16	0.14	50.68	60.41	+9.72
	8 × 8	**0.13**	50.89	**61.08**	**+10.18**
Imagenette (512 × 512)	32 × 32	0.13	79.79	83.45	**+3.66**
	16 × 16	0.13	81.20	84.26	+3.07
	8 × 8	**0.12**	81.15	**84.71**	+3.55

**Table 6:** Comparison of results for BCS [8], BCS-PCT [9], and BCS-asymmetry [10] on KITTI [51].

Average CS Rate	Sampling Technique	CS Rate			Reconstructed Image Quality		Detection Accuracy			
		**Saliency ($r_{\mathrm{high}}$)**	**Non-Saliency ($r_{\mathrm{low}}$)**		**PSNR [dB]**	**SSIM**	**Precision**	**Recall**	**F1-Score**	**mAP**
1.00	Original image		1.00		-	-	0.752	0.879	0.796	0.804
0.20	BCS		0.20		27.41	0.85	0.740	0.849	0.783	0.768
	BCS-PCT		0.20		31.02	**0.91**	0.745	0.861	**0.792**	0.784
	BCS-asymmetry		0.20		31.32	**0.91**	**0.746**	0.860	**0.792**	0.784
	Ours	0.50		0.05	**31.51**	**0.91**	0.737	**0.871**	0.790	**0.794**
0.17	BCS		0.17		27.41	0.85	0.740	0.849	0.783	0.768
	BCS-PCT		0.17		30.93	**0.91**	**0.746**	**0.861**	**0.793**	**0.784**
	BCS-asymmetry		0.17		**31.26**	**0.91**	**0.746**	0.860	0.792	0.783
	Ours	0.50		0.01	27.60	0.83	0.723	0.834	0.767	0.751
0.16	BCS		0.16		27.41	0.85	0.740	0.849	0.783	0.768
	BCS-PCT		0.16		30.89	**0.91**	0.743	0.859	**0.790**	0.781
	BCS-asymmetry		0.16		31.24	**0.91**	**0.744**	0.859	**0.790**	0.781
	Ours	0.40		0.05	**31.26**	**0.91**	0.736	**0.870**	**0.790**	**0.796**
0.14	BCS		0.14		26.29	0.82	0.724	0.813	0.757	0.728
	BCS-PCT		0.14		29.85	0.88	0.731	0.843	0.777	0.761
	BCS-asymmetry		0.14		**30.22**	**0.89**	**0.737**	**0.846**	**0.781**	**0.765**
	Ours	0.40		0.01	27.47	0.83	0.727	0.832	0.769	0.753
0.13	BCS		0.13		26.29	0.82	0.724	0.813	0.757	0.728
	BCS-PCT		0.13		29.80	0.88	0.731	0.842	0.776	0.761
	BCS-asymmetry		0.13		30.19	0.89	0.736	0.845	0.780	0.763
	Ours	0.30		0.05	**30.90**	**0.91**	**0.737**	**0.874**	**0.792**	**0.796**
0.11	BCS		0.11		26.29	0.82	0.724	0.813	0.757	0.728
	BCS-PCT		0.11		29.67	0.88	0.731	0.841	0.775	0.760
	BCS-asymmetry		0.11		**30.11**	**0.89**	**0.736**	**0.846**	**0.780**	**0.765**
	Ours	0.30		0.01	27.27	0.82	0.724	0.830	0.767	0.750
0.10	BCS		0.10		24.76	0.76	0.711	0.718	0.706	0.637
	BCS-PCT		0.10		28.36	0.85	0.733	0.788	0.752	0.715
	BCS-asymmetry		0.10		28.78	0.86	0.731	0.794	0.755	0.719
	Ours	0.20		0.05	**30.22**	**0.90**	**0.741**	**0.871**	**0.793**	**0.792**
0.07	BCS		0.07		24.76	0.76	0.711	0.718	0.706	0.637
	BCS-PCT		0.07		28.07	0.84	0.730	0.784	0.748	0.705
	BCS-asymmetry		0.07		**28.59**	**0.85**	**0.733**	0.786	0.751	0.714
	Ours	0.20		0.01	26.90	0.81	0.721	**0.824**	**0.762**	**0.743**
0.06	BCS		0.06		24.76	0.76	0.711	0.718	0.706	0.637
	BCS-PCT		0.06		27.92	0.84	0.728	0.782	0.747	0.704
	BCS-asymmetry		0.06		28.49	0.85	0.732	0.788	0.752	0.716
	Ours	0.10		0.05	**28.81**	**0.88**	**0.738**	**0.868**	**0.790**	**0.792**
0.04	BCS		0.04		22.15	0.66	0.658	0.458	0.525	0.387
	BCS-PCT		0.04		25.48	0.77	0.692	0.606	0.633	0.531
	BCS-asymmetry		0.04		**26.07**	**0.79**	0.699	0.627	0.650	0.550
	Ours	0.10		0.01	26.04	**0.79**	**0.723**	**0.802**	**0.753**	**0.723**

## Data Availability

Dataset available on request from the authors.
